# The Chicago Dental Society

**Published:** 1889-03

**Authors:** 


### The Chicago Dental Society.

REPORTED FOR THE DENTAL REGISTER
BY "MRS. M. W. J."

The twenty-fifth anniversary of the Chicago Dental Society was celebrated by a three day's meeting, held in the Grand Pacific Hotel, which was general headquarters for the society and its guests. Exhibits were made in the same building by the S. S. White Co., the American Dental Manufacturing Co., and others. Clinics were given at the Chicago Dental College.

The meeting was called to order shortly after 10 A. M. Tuesday, February 5, the President, Dr. J. A. Swasey, in the chair.

The meeting was opened with prayer by the Rev. G. C. Lorimer, of Chicago.

The President after a brief address of welcome introduced the reader of the first paper,

*Dr. A. H. Thompson*, Topeka, Kansas. The subject of his paper was

GUM-COLORED PORCELAIN FILLINGS,

Illustrated by a number of specimen fillings which were passed around for examination. He said that the art was yet in a crude state, though it had been employed in various forms for many years, especially in anterior visible surfaces of teeth. The special application to which Dr. Thompson wished to direct attention, was to the restoration of cavities in the necks of teeth.

exposed by recession of the gum. The highest art is to restore so that the restoration is not perceptible. In such cases this can be done by the perfect adjustment of a carefully selected portion of porcelain gum from artificial teeth, grinding to the proper thinness from the body side and allowing it to project sufficiently to give the natural prominence of gum. If the cavity extends into the crown of the tooth an inlay including both gum color and proper tooth color must be selected and ground to fit. Dr. Thompson said that he believes that though the inlay system is in its infancy it has a great future—that the porcelain filling is the filling of the future.

*Dr. C. Thomas*, Des Moines, Iowa, who was appointed to open the discussion of this paper, said that he scarcely knew how to commence as the method employed by Dr. Thompson was so very different from his own practice, which is to burnish into the cavity—no matter what shape it may be, platinum ribbon to form a matrix in which he bakes the tooth-body, making a porcelain inlay to fit the cavity, instead of grinding a piece from a porcelain tooth as described by Dr. Thompson. He uses this method for all cavities, restoring corners of teeth, building up molars, etc.

*Dr. Dorrance*, of Michigan, thought the method of Dr. Thompson more available than that of Dr. Thomas, the ready made teeth having better color and greater density than inlays baked in a matrix of platinum, though it requires greater skill to adjust a piece of broken tooth. He does not, however, believe either method universally applicable, but it is a subject well worthy our thought and best efforts, the material being calculated to do better service than gold or any other metal, being a non-conductor and like the tooth in appearance.

*Dr. Reid*, Chicago, wished to ask how the pieces were retained that were used in restoring broken corners, etc?

*Dr. Thomas*—Replied that a platinum pin was baked in the inlay, and inserted in a hole drilled in the tooth, the whole being retained by cement.

*Dr. Dorrance*—Described a large porcelain restoration in a central incisor. The tooth was alive, and very sensitive under gold fillings, but with the porcelain restoration the sensitiveness had entirely disappeared, and after four years the work is perfect.

*Dr. L. L. Davis*, Chicago, said that he had seen a number of porcelain fillings with platinum backings made by Dr. Land, but if they were fair samples he would not want them in his own mouth, nor would he recommend them to his patients. In one case, all the anterior teeth had been in such bad condition that all the enamel had been replaced by Dr. Land's method, but the result was disgusting, the shading was *way off*, no two pieces were alike, the lines between the patch and the tooth looked as if a dirty thread had been tied around each tooth. He did not think it either as good or as strong as a piece of artificial tooth would have been.

*Dr. Thompson*, the essayist, asked that some one who had used gum-colored porcelain, as described in the paper, would give his views on that point. No one replying

*Dr. Abbot*, Iowa, asked Dr. Thompson how he manipulated his pieces to retain them. Did he use dovetail or under-cuts? How were they retained in the cement?

*Dr. Thompson*—Replied that when he used two pieces they were dovetailed, and even with one piece there could always be an undercut on one end and one side, which, with good cement would hold very securely. It is an erroneous impression that cement is not durable. Though used right at the margin of the gum the secretions have no effect, because the crevices are so very minute that no moisture can enter, and then it can always be readily repaired with more cement. The effect of cement is to exclude moisture as it clings to tooth substance, and in conservative properties it is superior to any other filling material.

*Dr. Peterson*—Said that he was much interested in this subject as a member of his family had extensive caries at the necks of the anterior teeth where large gold fillings would be unsightly and a vulgar advertisement. He had inserted pieces of English teeth and having been fortunate in getting a good color-match, the work had given him great satisfaction. He thought it much more simple to use pieces of tooth than to fire up a furnace and bake new inlays.

*Dr. Fernandez*, Chicago, has used "English tips" which are made of good body and have a centre pin. They are held on wood attached by shellac and ground approximately to fit the cavity which is also shaped properly—after cementing in with oxyphosphate they are ground in proper contour.

*Dr. Thompson* said that with proper care an almost microscopical fit may be obtained.

*Dr. Sudduth*, Philadelphia, said that often the tip is quite closely adapted, emery is put in the cavity and the tip, held in a mandrel, ground in the cavity it can be fitted perfectly like a glass stopper in a bottle—the cavity of course being a round one.

*Dr. Harroun*, Toledo, said it was quite apparent that Dr. Thompson was entitled to all the credit of using gum-colored porcelain fillings as described in the paper, but that we all have occasional cases where they might be used to advantage.

*Dr. Green*, New Albany, has found that if the porcelain filling is heated as hot as it can be held in the fingers the cement will set much harder than by any other method.

*Dr. Fernandez*—Varnishes the cavity with sandarac. In cementing the inlay in he dries the cavity thoroughly, and uses his cement very thin at first, adding it thicker till the last is quite thick; the inlay is pressed firmly home in this, and after ten or fifteen minutes the porcelain is ground to contour.

*Dr. Pruyn*, not being present to read his paper, next on the programme,

*Dr. T. E. Weeks*, Minneapolis, read the paper appearing on the programme as

*Obtundents of Sensitive Dentine,*

but which he desired to change to

SENSITIVENESS AND ITS CONTROL.

After describing the structure of the tooth tissues and its surroundings, the function of the pulp, and the condition known as Sensitive Dentine, the writer describes the various methods adopted for relieving this painful condition. De-hydration, either by raising the temperature as by hot air, or lowering it by evaporation; by the rapid motion of a keen instrument. He also dwelt upon the importance of securing the confidence of the patient, the influence of mind upon mind, and the effects of imagination.

*Dr. L. Custer*, Dayton, Ohio, who was appointed to open the discussion of this paper, said that his ideas in general conformed to those of Dr. Weeks, especially in regard to the part played by the imagination, the cultivation of which makes it more vivid. The suffering produced by the fear of pain is as intense as that caused by the actual pain. Sight, hearing, and smell all play important parts, and every thing having painful associations should be avoided as far as possible.

Obtundents produce their effect either through change of structure—as in the action of coagulants, or through the reduction of temperature, as in dehydration, or through withdrawal of nutrition.

The various combinations of these methods will often be more valuable than any one alone. Much may be accomplished by the intelligent use of instruments alone, without any medicaments.

*Dr. W. H. Atkinson*—Congratulated the society on the erudition of its speakers and said that the oldest present could listen with benefit and instruction to the young men of to-day who have stolen all the honey from the comb of the old fogies.

All that we know is but a mode of consciousness. We know pain only by the uncomfortable movement of what we call consciousness. Our affection for our patients is at the root of all we can do for them, and we should not attempt to operate for those with whom we are not in sympathy.

*Dr. A. H. Thompson*—Prefers, where the dentine proves to be very sensitive, to put in a temporary filling of oxyphosphate and wait a week or two.

*Dr. Harlan*—On the other hand finds sensitiveness accelerated by this mode of treatment, due he thinks, to excess of any uncombined phosphoric acid. It is only after two or three months that it is dissipated. He said that, with the best of motives he had put forward a number of agents that he thought he had found valuable in obtunding sensitive dentine, but no one or two or three remedies have universal application.

Desiccation when practicable, and if not carried too far, is very frequently useful. The anæsthetic essential oils which permeate the dentine are more valuable than the coagulants.

*Dr. A. E. Baldwin*—Uses very sharp instruments with a light touch and rapid motion.

*Dr. J. Taft*—Said that it was very important to understand principles—then correct practice would be likely to be adopted. All cases of sensitive dentine are not alike, and are due to a variety of influences, peculiar to the individual, modified by the tissues, by the constitution. An enfeebled patient in less than usual health and vigor, not well-nourished at the time, whose system and tissues are easily irritated, will be especially liable to suffer from sensitive dentine. There are different degrees of sensitiveness, from that which is barely appreciable to that which is intense and well nigh unbearable; it may be only a superficial layer, or a small spot, or it may permeate the dentine of the entire crown of the tooth. All these peculiarities indicate different modes of procedure. The *expectant method* is quite practicable; gutta percha or Hill's stopping is preferable to oxyphosphate as a temporary filling until the tone of the system can be restored. After thorough drying of a cavity with bibulous paper the use of absolute alcohol evaporated by warm air followed by saturation with creosote, oil of cloves or cinnamon, etc., with a jet of hot air on that, will relieve many cases of sensitiveness.

*Dr. Chas. Pruyn*, Chicago, next read a paper entitled,

### A STUDY OF THE EFFECTS OF COCAINE UPON MAN AND SOME OF THE LOWER ANIMALS.

He said that cocaine should be used with the greatest discretion as its abuse is likely to lead to the cocaine-habit, which is incomparably worse than alcohol or opium in fascination and seductiveness, and it is one of which when once formed the demands will not be denied. When you think of using it in dental surgery think of this possible danger.

No man ought to begin its use in his practice until he has studied its effects upon the lower animals even to death. In that way only can he understand the symptoms, recognize their indications, and know what to do.

The general surgeon can hold the circulation suspended and restrain the action of cocaine to the point operated on by the use of Esmark's bandages, tourniquets, etc.,but this is not open to the dental surgeon who must therefore use much less than is permitted with safety to the general surgeon.

The dental surgeon injects it into tissues near the main nerves and grand arteries and his risks are much more serious. Three drops of a four per cent. solution injected into each side will usually be found sufficient for the extraction of a tooth ; great care must be taken that none be swallowed, or nausea will follow. It will be found that cocaine acts best on dense fibrous tissues, loose, flabby tissues being more difficult to anæsthetize. The sensations most usually experienced are a sense of rest and peace, the patient seems to be in a world of ecstatic bliss. The toxic symptoms are great increase in the rapidity of respirations with decrease in depth, faintness, palpitation, stifling, lack of air, tingling of the extremities followed by cold, involuntary muscular movements, and convulsions if not checked.

The heart beats after the respiration ceases. Paralysis of respiratory centres is combatted by injections of brandy or ammonia, the inhalation of nitrate of amyl. Thirty drops of aromatic spirits of ammonia may be given to swallow. If morphine is to be used as an antidote it should be injected some thirty minutes before the cocaine, and not after toxic effects are manifest. Given in combination only the effect of cacaine will be perceptible.

Dr. Pruyn then gave the history of a long series of experiments with cocaine on dogs which seem to prove conclusively that opium is an antidote to cocaine, dogs which recovered entirely from the effects of a certain dose of cocaine when antidoted with morphia and atropia, dying from a subsequent administration of the same dose of cocaine without the antidotes.

Dr. Pruyn's record of cases in office practice shows that a very small amount of a weak solution in the stomach produces nausea and vomiting. The four per cent. solution in small doses is best adapted to dental operations, the seven and eight per cent. solutions producing toxic effects. While physicians use from five to eight grains in general practice one grain is a large amount for alveolar injection, and the fifth pair of nerves being more impressionable to the action of cocaine than any other part of the system.

*Dr. J. B. Morrison*, Indianapolis, who was appointed to open the discussion, said that the essayist had covered the ground so completely that he had left nothing for discussion. In his own practice he had confined himself entirely to its topical effects upon the gum for lancing, tying ligatures, etc.

For this purpose he uses it in a very strong solution—almost a syrup of the crystals, applying it on a shred of cotton.

Painting the nostrils in the same way will almost invariably break up a cold in the head, a second application being seldom required. For extraction he considers ether or gas both safer and surer.

*Dr. Martindale*, Minneapolis, has used it with perfect satisfaction since Jan., 1885. He has not found the exhilarating effects mentioned by Dr. Pruyn, and thinks that much is due to the imagination as also the toxic effects which can be apparently induced by adroit *questioning* of the patient, leading the mind in that direction.

*Dr. Atkinson*—Said that all these experiments only show an entire lack of uniformity in results; the personal equation must be taken into account to a degree of which we had no conception.

*Dr. Wm. Conrad*, St. Louis, said that cocaine in the hands of many has been much abused; in his own practice he had had no bad results, no alarming symptoms. He uses from one-quarter to one and-a-half grains. But the more he hears about it, the more cautious he becomes. We can not do without it, but we must use it with proper precautions. Dr. Martindale does not like the addition of acids to prevent decomposition but prefers to dissolve the crystals at the time of each operation.

*Dr. Pruyn*—Said that by the addition of either carbolic, or salycilic acid he has used the same solution for four months without any deterioration—kept in open daylight but not directly in the sun. The cases in which *not* to use cocaine are fatty degeneration or any other form of heart trouble, any form of lung trouble, any form of kidney trouble, in an exhausted condition of the nervous forces, in pregnancy.

Its results are always doubtful as it is the most uncertain drug that we have to deal with. In its use know your patient well, and have all known remedies at hand. It is a valuable remedy but should not be abused.

*Dr. J. J. R. Patrick*—Read a paper on

THE STUDY OF PRE-HISTORIC REMAINS IN THEIR RELATION TO DENTISTRY.

He spoke of the great tribes of Indians that roamed over the western prairies previous to the visits of Marquette, Joliet, Hennepin, La Salle, etc., and even up to the date of the surveying and platting of the present city of Chicago, and whose remains by thousands still lie buried beneath the soil, ready for that scientific study and investigation from which valuable results may be obtained if undertaken in the right spirit. In any one branch of science marked advances are made only when connected with the progress of some collateral science. The astronomer and the optician together make rapid progress. As a mere matter of curiosity it was long known that soft amber rubbed would attract like substances, but time brought the theorist and the machinist together and what triumphs they have achieved!

So of chemical science and the study of the hard tissues of the body; of the microscope and the science of physiology; of morphology and the investigation of the structure of plants. The theoretic man must have manipulative skill. It is not what a man sees but how he sees it. What he has learned furnishes the substructure for systems of thought. The senses are teachers but they must be disciplined and trained to be reliable. The division of labor is the main principle in advancement. The rapid advancement of our specialty is largely due to the work of associations, the combination and comparison of the observation of many separate workers, but care should be taken not to give undue respect to superficial observers to unverified observations; our methods are too effusive, and we should be more exact.

So if the question before us—the comparative frequency of diseases of the teeth of civilized and of savage man—to judge properly of this we must be able to answer the question--Was the dental system of uncivilized races free from anomalies? Free from the diseases of the oral cavity so common to civilized man? The average practitioner will say without hesitation, the savage was exempt from all that; in civilized man it is due to artificial modes of life, mental capacity increases at the expense of the physical, etc.

But if statistics are called for we have them only from the side of civilized life. The savage had no physicians or dentists whose records we may interrogate--hence the conclusions drawn are one-sided and not scientific. But it is not too late to put scientific workers in the field for the study of the still abundant remains of pre-historic man.

They are to be found in our museums and thousands are yet undisturbed beneath our soil. The subject is yet in comparative obscurity but it is one of great importance and one which grows in interest as it is investigated.

*Dr. McKellops*--Said that several years ago he had offered a resolution in the American Dental Association calling for an appropriation of money for the investigation of this subject, but nothing had been done. The paper shows the great need of this work and the proper men, such as Dr. Patrick, should be put in the field, under conditions that will enable them to give it their best skill and thought.

*Dr. Atkinson*--Says it is self-evident that sufficient investigation has not been made even to entitle us to an opinion on the question--not even a hypothetical solution.

*Dr. E. T Darby*, Philadelphia, says that while we do know something about pre-historic teeth, we do not know half as much as we ought to. He has given some study to the subject especially the skulls from ancient tombs of the old world; from these we know that the ancients were subject to many of the conditions that affect us at the present day.

With his own hands he had dug out mummies from the mummy pits as they were opened, and found the teeth of two and three thousand years ago, immeasurably better than would be the case with any similar number of modern skulls; in fact, in all those skulls he found but one missing tooth, and two cases of caries. He had also examined the teeth of the skeletons in the Convent of St. Catherine, Mt. Sinai, which for fifteen hundred years have been ranged in tiers, in the order of the centuries. With but rare exceptions he found no caries in the teeth of the most remote remains, but marked increase in those of comparatively late date. The teeth of the Bedouins of the desert are much better than those of the inhabitants of cities. Their teeth are good except in abrasion from the hard food used and sand and ashes mixed with it in their primitive modes of cooking. The Syrians, who use more animal food, have poorer teeth than the Bedouins of the desert who use more phosphatic food.

*Dr. A. H. Thompson*—Said that twenty or even ten years ago it was usual to claim that the savage had better teeth than civilized man because of his more natural mode of life, but all that is exploded now. The savages that had weak teeth and weak health died early and their failings were not perpetuated, while we preserve the weakest with the greatest care. Some years ago he had brought out the idea that the teeth of man are in course of suppression—of this we have evidence in the case of the wisdom teeth. This is related to the question of the effects of use and disuse, but has nothing to do with the fact that we are better and stronger—live longer and better than the savage did.

*Dr. W. X. Sudduth*—Said there could be no question of the necessity and value of tabulated observations on this question. It was a shame that the profession should rest satisfied with the superficial view heretofore taken. We discuss the questions of the influence of civilization and of diet upon the teeth without any just basis for argument.

It is the especial prerogative and the duty of the dentists of Illinois with the vast material of the mounds within their territory, to take hold of this subject and bring out the facts. Many years ago a committee had been appointed to investigate the subject, but the matter had died there. From a utilitarian point we want broad comprehensive views. The men best fitted for this work should be financially supported in it.

*Dr. Crouse*--Said that he felt that he had been hit in all this as he was chairman of the committee so often referred to. That was in the old days when they were all jealous of each other, etc., but he thought they would soon wake up again and would be heard from.

*Dr. Patrick*—In closing the discussion said that what we want is positive knowledge and scientific methods. Swearing "to the best of our knowledge" may be legal but it is not scientific. If a Tyndal were to go before his class and say that he had demonstrated to his own satisfaction that gold is not an element, but is composed of two elements, but that he could not demonstrate it, his word alone would not be accepted. We can not be slipshod in matters of science. Before we can accept what a man says he has seen we must see an optician and find if his eyes are sound. We must have demonstrated facts; stubborn irresistable evidence must be given. At present, on this question, the Indian is only a scapegoat on whom we cast all the anomalies and diseases of civilization.

Dr. J. H. Martindale, Minneapolis, read a paper entitled,

### CARIES AND NECROSIS IN THEIR RELATION TO PRACTICAL DENTISTRY.

When inflammation has been induced in the maxillæ or alveolar appendages by traumatism, septic infection, cachexia, or specific mineral poisons proceeding through the stages of congestion and stasis, nutrition being cut off *necrosis* must result. But if the blood current has been merely slowed the bone becoming the seat of perverted nutrition with both reconstructive and destructive elements present we have *caries* of bone.

*Nercosis* is a condition analogous to gangrene in soft tissues, *caries* being similar to that of ulcerations. In *caries* a molecule dies and is carried off in the death waste, a healthy molecule right beside it waging valiant war against the usurper; in *necrosis* the defending forces are like besieged soldiers cut off from all sources of supplies; they surrender the barracks leaving it a mere mass of dead matter.

The periosteum covering necrosed bone is often lifted up by the effusion of inflammatory products, a film of new bone forming beneath which invaginates the dead bone and which must be cut through for the removal of the latter. If in the lower jaw the effusion between the internal and external plates bulging out the latter forming a tumor-like appearance. In some cases an outlet is formed and the plate collapses; in others new cells are proliferated and a permanent tumor results. Among the predisposing causes are the exanthimatious diseases common to childhood—measles, scarlet fever, chicken pox, etc., (the alveolar process involving the deciduous teeth and even the follicles of the permanent ones sometimes being exfoliated), inflammation aroused by traumatism, especially in scrofulous or tuburculous diathesis, constitutional syphilis, mercurial poisoning, the inhalation of phosphorous fumes, etc. Diseased conditions of the maxillæ may also originate in diseases of the teeth and in ill-advised or careless dental operations; among the former dead pulps, encysted teeth, erupting wisdom teeth; among the latter heavy malleting, violent wedging, and improperly filled roots; also the application of arsenious acid; many cases are also undoubtedly idiopathic.

In the outset diagnosis is obscure, the indications being similar to those of periostitis and the pain often described by the patient as toothache.

Aromatic sulphuric acid is the main reliance in treatment conjoined with injections of peroxide of hydrogen, surgical aid being frequently required in the removal of sequestra.

Dr. Martindale concluded his paper by the recital from his case-book of a number of typical cases.

*Dr. Atkinson* said he did not know how to express the effect produced upon his mind by the reading of the paper better than to say that if he had heard it read in a foreign land as one of "Atkinson's papers" he would certainly have accepted it as his own, it was so almost axactly the statement of his own methods. The processes through which the tissues pass under the stress of inflammation are uniform. They are swollen and congested, melting down till granules of embryonic tissue are separated constituting puss; a step beyond this we get sanies, and with increasing virulence, ichor.

Through the action of aromatic sulphuric acid on necrosed bone the tribasic phosphate of lime is changed into the bibasic phosphate which crumbles and is more easily taken away. It coagulates the surface of the healthy portion forming a scab that is thrown off, proliferations of granulating tissue appearing beneath like a scab from any escharotic which does not vesicate.

*Dr. Atkinson* said that he wanted to repeat what he has taught in regard to the sponge graft which if properly used favors reproduction of tissues. A delicate impression should be taken and the lost portions built up in wax making it a little flush as the reproduced tissue will shrink. Gum tissue is a succession of blood sinuses proliferated from its own walls and when reproduced defies detection. A sponge graft should always be protected by a delicate platinum shield.

*Dr. Brophy* said that caries of the alveolus usually had its origin in an alveolar abscess from a dead pulp, the discharges through the foramen softening the bone around the apex of the root. The action of aromatic sulphuric acid is not always sufficiently rapid and often requires surgical interference. An incision should be made sufficient to expose the diseased parts; the wound should be packed with boracic acid gauze which gives a better effect than iodoform and obviates the odor. The next day a thorough examination should be made with a long-drawn-out, flame-shaped bur the diseased roots which interfere with the formation of new tissue should be cut off and with a bud-shaped bur all carious bone removed. Packing the wound with crystals of boracic acid, dismiss for two days when it is probably that red eyes will be seen shooting out, the effort of nature to close the wound. As soon as this is observed the wound should be plugged with wax softened and moulded to fit the cavity which will not absorb or ferment and which must be shaved down as the wound heals from the inside until no longer needed.

*Dr. Martindale* in closing the discussion said that he had tried the sponge graft faithfully but always in vain the result being invariably a filthy mess. He often experienced trouble from packing wounds in patients failing to return at the proper time after which the pressure of the packing will break up granulations.

Dr. Hanson was introduced by Dr. Haskell. He spoke at length on the beneficial effects of *heat* as a curative agent.

*Dr. G. V. Black* read a paper embracing the results of a series of experimental tests of the value of

ANTISEPTICS.

He said that the antiseptic value of a drug is best expressed by its range of effective work. This range of value it found in the difference between the saturated solution, or that of concentration that may be found injurious to the tissues, and the greatest dilution that inhibits the development of micro-organisms. Those essential oils that are not too irritating have an extension of range in their use in emulsion, or in substance. Also many drugs have, in greater dilution than that which actually inhibits, a range of restraint that is useful.

The essential oils are growing in favor but there is a lack of exact knowledge concerning their peculiarities. Many that are held in high esteem are not antiseptic at all, while others that are scarcely in use have very valuable antiseptic qualities. His experiments prove conclusively that iodoform has no antiseptic value, micro-organisms developing in the undissolved powder in the bottom of the test tube used in the experiments. This also occurred in the emulsion of the oils of cajeput, thyme, wintergreen, and several other of the esteemed essential oils, among the inhibiting oils being the oils of cassia, cloves, cinnamon, mustard, peppermint, sassafras, and others. The great difficulty in the use of the essential oils lies in their uncertain qualities, being usually prepared in the locality where the plant grows by unscientific workmen with probably a careless mingling of different parts of the plant possessing very different qualities. As their value becomes known we shall probably be able to secure their preparation in a more worthy manner.

In the experiments of Dr. Black peptonized beef broth, infected with his own saliva as far as possible under the same conditions was used as the culture medium, the tubes graduated to secure accurate measurements, and kept five days in an incubating oven at a temperature of 99°, unless growths occurred earlier. The oils were tested both in substance and in solution.

The aqueous solutions were made by violently shaking together, placing in the oven twelve hours, shaking again, and after a second twelve hours in the oven repeatedly filtered until perfectly clear. Every precaution was taken to secure exactness, every experiment being repeated until no doubt was left as to the result. One fact to be borne in mind is that the greater the range of inhibitory value the greater the toxic effects; if not poisonous their range of value is very short. Thus eucalyptus is very feeble, only a saturated solution being effective, but at the same time it is perfectly safe having no toxic effects even by absorption. Bichloride of mercury which on the other hand has the greatest range of value, is also the most poisonous. Oil of cassia holds its own in comparison with carbolic acid, boracic acid, or resorcin. The pure oil blisters the skin, healing, however, perfectly beneath the blister; in emulsion, except in very large wound surfaces it gives excellent results in preventing pus formation. It can be largely diluted with bland oils and give good results—"1–2—3" or

| | |
|---|---|
| Carbolic acid - - - | 1 |
| Oil of cassia - - - - | 2 |
| Oil of wintergreen - - | 3 |

Will be found excellent, having an antiseptic range greater than carbolic acid alone without its evil effects.

Antiseptics are used in three forms; in water for washings, the oil, and in powder. Washings can not be made continuous, therefore, limited in usefulness in dental practice. Solutions in peroxide of hydrogen have a greater value then in water, as we have the added effect of the mechanical liberation of oxygen carrying the effects into the most remote parts. The oils are more effective from their diffusibility and remaining in position longer, doing their work continuously. They are especially applicable in the treatment of root canals where they will do good for many days together. Oil of cloves and cinnamon are less distasteful than some others. Mustard is too irritating but is very valuable as a stimulant and is the most diffusible of all the oils. An emulsion of any of the oils may be made by shaking violently in a bottle with water and passing through a syringe a number of times.

Boracic acid stands at the head of the list for use in powder, as a dry antiseptic dressing. Hydronaphthol may be used in the same way but is not so well borne by the tissues and has less antiseptic value. Dry powder dressings are liable to cake and form an arch under which septic conditions may exist; this must be guarded against. A combination of oil and dry dressing gives enhanced value to both.

The fourth form, more rarely employed, is that of hypodermic injections where as in stumps, tumors, erysipelas, gangrene, etc., it is desirable that the tissues should be injected with the remedy. Dr. Black states that he had once prevented the amputation of a gangrenous stump higher up by injecting the tissues very completely with salycilic acid. It sloughed off with no recurrence of gangrene. It is found that liquids diffuse very slowly through contracted openings—as of abscesses and sinuses—hence the value of peroxide of hydrogen, and from this fact also we learn that the action of medicaments in a root canal will not extend beyond the apex unless forced through when applied.

The discussion of this paper was opened by *Dr. C. M. Bailey*, Minneapolis. He said that the distinction drawn by Dr. Black between antiseptic and disinfectant was valuable and not generally recognized. It should be borne in mind that antiseptics inhibits the growth of micro-organisms, but does not destroy them when already present, while a disinfectant so-called destroys them. The term germicide should be substituted for disinfectant as conveying the full significance. In the selection of the agent we must consider both its power of control over micro-organisms and also its action upon the tissues. It must not be forgotten that destructive metamorphosis of tissues is largely due to a lowering of the vital forces of the system. Microbes have no power of growth when the vital powers are strong. The system drives them out of itself without the aid of drugs, as is seen in a common boil.

In regard to iodoform, while laboratory tests prove that it has no antiseptic value, yet in clinical practice we find that it does prevent the formation of pus; it therefore has a use in the prevention of disease and we continue to use it until we find something that will do it better. We find too that eucalyptus oil and iodoform will effectually sterilize the worst root canals with discharging abscess. These remedies may be powerless but the microbes succeed. We therefore use them though empirically.

*Dr. Sudduth* said that Dr. Black, by this synthetic method, presented facts of incalculable value. He criticised the method of infection adopted, the saliva even of the same individual being so varying in character. He thought it quite practicable to thoroughly disinfect the oral cavity.

He considered the use of essential oils in a tooth cavity objectionable as liable to affect the integrity of the filling. Iodoform used as a dry dust dressing by surgeons seems to undergo some chemical change by contact with the secretions by virtue of which it does exhibit valuable properties not yet explained. In opthalmology boracic acid has beed discarded in favor of silica-fluoride of soda.

In the disinfection of pulp canals bichloride of mercury is undoubtedly the most effective agent we have; where a stimulant effect is desired Dr. Black's "1—2—3" is most excellent; where a non-poisonous non-irritant is essential we may perhaps find it in the silica-fluoride of soda. It is not generally known that oil of cloves will prevent the disintegration of the rubber dam; it has lasted for five years as a stopper for drop-bottles when without this preparation it rots in less than six months. This is a practical point worth knowing.

*Dr. A. H. Thompson* has found in his practice that bichloride of mercury turns a tooth very black.

*Dr. Harlan* expressed his gratification at finding his views as to the value of the essential oils so confirmed by the tests of Dr. Black. Their value has long been overlooked except among household remedies.

The experiments of Dr. Black with the oils moistening a cotton stopper, the vapor alone inhibiting growth on the surface shows the great value of the deposited champhors. In infected dentine the vapors will be diffused throughout the dentine with an effect that can be obtained by no other method.

*Dr. J. H. Wooley* has found that after the most thorough drug disinfection, a heated broach will still reveal by the odor of suphuretted hydrogen, the presence of putrefaction which can only be destroyed by repeated applications of heat; this is best done by means of a heated broach.

In closing the discussion Dr. Black said that the whole subject is young as yet; as we grow in knowledge we may have to take out whole blocks from our foundation and build anew. Flaws may be found in our technique to prove our results all wrong; but we will continue to work at it.

WEDNESDAY EVENING.

We hope to give this paper in the April number. *Dr. R. R. Andrews* read an interesting paper, and gave a lantern exhibit of photo-micrographs showing

THE DEVELOPMENT OF TEETH, THE FORMATION OF DENTINE AND ITS APPEARANCE IN HEALTH AND DECAY.

The first picture projected upon the screen showed a section of the lower jaw of a human fœtus, showing the embryonal tissues with the slight depressions, marking the place of the future teeth. The succeeding illustrations showed the gradual development of the tooth through all its stages, in the human jaw or the pig. In the tenth the papilla was shown well advanced, with dentine-cells and the ameloblastic layer. In the eleventh the tooth shape is well developed, the cells on the edge are changing to odontoblasts, and the bud for the enamel organ of the permanent tooth is seen on one side. In the fourteenth was seen the breaking up of the cord of the temporary tooth, the bud of the permanent going down under the other. The fifteenth showed the tooth at the eighth month—dentine and odontoblasts, the enamel forming, cementum, tissue, etc. The eighteenth—one of Dr. Sudduth's specimens—showed the line of ameloblasts or Tomes' processes a band of formed dentine, and the tissues of the pulp of the tooth. The next, the odontoblasts, under very high power, the fibrils.

The twentieth showed tracts of calco-globulin, a peculiar formation called by Dr. Andrews "the borderl and of calcification"—small globules or calco-spherites, of glistening appearance like fat cells, laminated in structure like tiny onions. This formation was also seen in Nos. 23, 24 and others. No. 22 shows a cross section of normal healthy dentine, and also a long section looking in at the open ends of the tubuli. The next series showed forming and formed dentine in varying stages. The thirty-first shows the origin of the interglobular spaces in dentine, with a band of uncalcified or calcoglobulin imprisoned between. 32. A pathological specimen, the tubules enlarged by the presence of organisms found in the depths of dentine.

Dr. Andrews said that to Dr. G. V. Black he owed the suggestion that if micro-organisms could be photographed, it might do much good. He had therefore gone to work in that direction with the following results:

33. Streptococcus, little round bodies in chains, so small that it is almost impossible to see them. He had made thirteen attempts before getting this specimen.

34. Bacillæ found in superficial layers of caries—little long bodies.

35. Diplococci, found in deeper layers. These are the "little bugs" whose waste product is lactic acid, causing softening of tissue.

36 Also shows the organisms which cause decay of the teeth—micrococci, which cause the breaking down of dentinal tissue in caries.

38, 39, 40 and 44 Sections of carious dentine, with tubules distended and filled with micro-organisms, or broken down, the organisms getting into the anastomosing canaliculi and passing from one tubuli to another.

46 and 47 specimens of artificially produced caries, made in a tube with pure culture, showing the same distension of the tubes and crowding of tissue by their growth pushing into the oasis substance, with identically the appearance of natural caries.

51, 52 and 53 were still other illustrations of curious dentine and micro-organisms *in situ*.

*Dr. G. V. Black* who was called upon to open the discussion, said that after such a grand presentation it would seem most fitting that we should stop and think it over; that it was such a showing as he had not dreamed could be made. It was as though all had been looking through the one little glass, at once seeing microscopic objects which, until Dr. Andrews had got hold of this method, only the very few scientific workers in laboratories could see. The pictures are made from the slides, just as the sun printed them; made by light itself, we should accept them as light upon the subject of the most vital importance to us. After this presentation no one can doubt the presence of the organisms in carious dentine; as to their function each one can have his own opinion.

Dr. Black said that he had observed the formation of calcospherites or calcoglobulin in abnormal conditions in pulps, as in nodules; also, in varicose veins, in malformed teeth, where all is chaos, about the ends of long bones in young animals. We might expect some abnormality in all these positions, or when left to chemical forces in jars in the laboratory. Where life forces have not their perfect working, chemical forms result. This band of forming material pointed out by Dr. Andrews may not always be calcoglobulin, but may be an appearance due to post-mortem change. In the preparation of specimens, in the use of some reagents, we get this formation very frequently, while in other modes of work we do not get it.

Dr. Sudduth said that this is the subject nearest his heart of all temporal things. The work seen this evening has never before been accomplished. Dr. Andrews has done what no one before him has ever done--affording an occular demonstration of the close analytical work going on in the laboratories of a few--affording positive demonstration of the presence of micro-organisms in carious dentine. Each one is at liberty to form his own opinion as to their function, but their presence has been demonstrated. The literature on the subject is also open to all.

Dr. Sudduth said that in all his preparations he had never found the appearance pointed out as calcoglobulin on the border land of calcification. It has to him one of two aspects--either pathological or artificial. The specimens shown of normal structure show calcification taking place without that globular arrangement.

In closing the discussion Dr. Andrews said he was ready to place his specimens in the hands of Dr. Black and Dr. Sudduth to see what they would make of them. If they decide they are pathological he is willing to accept their decision.

Dr. W. L. Comstock--Indianapolis--read a paper on

ARTISTIC METHODS IN PROSTHETIC DENTISTRY.

This was an extension and continuation of the admirable treatment of the subject published in the January issue of the *Ohio Journal*, which was there illustrated by a manikin with movable diagrams, showing the results of neglecting to observe or to correct various irregularities, disproportions and other defects. The prosthetic dentist, to produce artistic work, should be able to see as the artist sees—he must compare the distorted face before him to which he is to restore its true proportions by means of an artificial denture, with the ideal outline of the human face. He should be able to handle charcoal or pencil at least sufficiently to indicate in detail the defects noted after careful study, and to embody the whole in a single sketch, not as a portrait, but as an indication of form and proportion; this should be both in profile and in full face. The *eye* should be trained to *see*, and the *hand* to *record* the actual, and the *mind* to *estimate* the relations and surroundings. The facial line, the angle of inclination, the contour of the face, the shape of the maxillæ, the angle of the lower jaw--all this should be noted and embodied in the sketches from which to construct the ideal, artistic piece of prosthetic dental workmanship.

This paper was briefly discussed by Drs. Dorrance, Edmonds, Allport, Haskell and others, the general conclusion being that, though eminently desirable, the methods of Dr. Comstock were scarcely practicable except to the few who are artists as well as dentists. In closing the discussion Dr. Comstock said that he considered it entirely practicable to have the methods of artistic adaptation systematized and taught, as it is all hand, on art principles. All who can learn to write can learn to draw. Art will add to the advancement of our profession as much as surgery has done.

Dr. D. L. McGraw, Mankato, Minn., had been announced for a paper on

### OBTUNDING SENSITIVE DENTINE AND THE CONTROL OF PERIDENTAL INFLAMMATION BY ELECTROLYSIS.

He was unable to be present, but, his paper having been received by mail, was read by the Secretary.

He said that notwithstanding the wonderful advancement made in the last few years, we were still open to the charge of causing untold suffering, which we seem incompetent to relieve. Especially is this true in our operations upon sensitive dentine, obtundents proving failures except in drawing dollars. Obtunding by electrolysis is not patented either in drugs or appliances. All the requisites can be purchased in open market; the method is safe; it can have no evil results, and it does all that is claimed for it. A 12 per cent. solution of cocaine in absolute alcohol, with 6 per cent. of alum is applied on a pledget of cotton; the positive pole being placed on the cotton and the negative pole on the cheek, a galvanic current—four cells being strong enough—is passed through for three minutes; after the same interval it is resumed for another three minutes, when sensibility will usually be found entirely overcome. Here, then, we have an agent that is willing to do our bidding and only waiting for our commands. No objection can be made to its use, while the results will prove most gratifying to both patient and operator.

Owing to the lateness of the hour this paper was not discussed, the hour for final adjournment having arrived.

---

MICROSCOPY.—Several years ago there was a general complaint that American makers of microscopes charge very much more for their work than the same kind of work could be purchased in Europe. There is no ground, however, now for such complaints. As cheap microscopes, and of much better quality, are made in this country as are manufactured in Europe. When a good American instrument can be bought for from $25 to $32, it seems to us that everything in the way of cheapness has been accomplished. Just think of a microscope, the quarter inch of which will resolve pleurosigma angulatum, made in this country and sold for $26.

